# Adipose-derived mesenchymal stem cells improve glucose homeostasis in high-fat diet-induced obese mice

**DOI:** 10.1186/s13287-015-0201-3

**Published:** 2015-10-31

**Authors:** Mingjun Cao, Qingjie Pan, Huansheng Dong, Xinxu Yuan, Yang Li, Zhen Sun, Xiao Dong, Hongjun Wang

**Affiliations:** Colleges of Life Sciences, Qingdao Agricultural University, 700 Chenyang Road, Chenyang, Shandong 266109 P.R. China; College of Animal Science and Veterinary Medicine, 700 Chenyang Road, Chenyang, Shandong 266109 P.R. China; Department of Pharmacology & Toxicology, Virginia Commonwealth University, 1 Hayden Dr., Petersburg, VA 23806 USA; Department of Surgery, Medical University of South Carolina, BSB 641, 173 Ashley Ave, Charleston, SC 29425 USA

## Abstract

**Introduction:**

Effective therapies for obesity and diabetes are still lacking. The aim of this study was to evaluate whether a single intravenous infusion of syngeneic adipose-derived mesenchymal stem cells (ASCs) can reduce obesity, lower insulin resistance, and improve glucose homeostasis in a high-fat diet-induced obese (DIO) mouse model.

**Methods:**

Seven-week-old C57BL/6 mice were fed a high-fat diet for 20 weeks to generate the DIO mouse model. Mice were given a single intravenous infusion of *ex vivo* expanded syngeneic ASCs at 2 × 10^6^ cells per mouse. DIO or CHOW mice injected with saline were used as controls. Body weights, blood glucose levels, glucose, and insulin tolerance test results were obtained before and 2 and 6 weeks after cell infusion. Triglyceride (TG), high-density lipoprotein (HDL), and insulin levels in serum were measured. Expressions of genes related to insulin resistance, including peroxisome proliferator-activated receptor γ (*PPARγ*) and insulin receptor (*InsR*), and inflammation (*IL-6,F4/80*, and nucleotide-binding oligomerization domain containing 2, or *NOD2*), were measured in livers at mRNA level by real-time-polymerase chain reaction analysis. Beta-cell mass in pancrheases from CHOW, DIO, and DIO + ASC mice was quantified. GFP^+^ ASCs were injected, and the presence of GFP^+^ cells in livers and pancreases was determined.

**Results:**

DIO mice that had received ASCs showed reduced body weights, reduced blood glucose levels, and increased glucose tolerance. ASC treatment was found to reduce TG levels and increase serum HDL levels. In livers, less fat cell deposition was observed, as were increased expression of InsR and PPARγ and reduction in expressions of IL-6 and F4/80. Treated mice showed well-preserved pancreatic β-cell mass with reduced expression of F4/80 and TNF-α compared with DIO controls. GFP^+^ cells were found in liver and pancreas tissues at 1 and 2 weeks after cell injection.

**Conclusions:**

ASC therapy is effective in lowering blood glucose levels and increasing glucose tolerance in DIO mice. The protective effects of ASCs arise at least in part from suppression of inflammation in the liver. In addition, ASCs are associated with better-preserved pancreatic β-cell mass.

## Introduction

Type 2 diabetes (T2D) is a complex metabolic disease characterized by insulin resistance and pancreatic β-cell destruction. With the improvement of the standard of living around the world, the aging of populations, and the increase in obesity, the incidence of diabetes has become a global epidemic [1]. Obesity is a leading cause of insulin resistance and T2D. Therapies that can reduce obesity and insulin resistance may reduce the onset of T2D [2].

Mesenchymal stem cells (MSCs) are adult stem cells that have multipotent differentiation ability. According to the description of MSCs from the International Society of Cellular Therapy, MSCs attach to standard plastic tissue culture dishes, express certain cellular markers (including CD90, CD44, CD105, and the absence of CD45, CD31, etc.,), and can differentiate into adipocytes, osteocytes, and chondrocytes [3]. MSCs can be harvested from bone marrow, adipose tissue, umbilical cord, dental pulp, fetal liver, lung, and many other kinds of tissues [4]. Adipose-derived stem cells (ASCs), which can be readily isolated from fat tissue after liposuction and easily expanded in culture in large numbers, have become an attractive source for cell therapy. ASCs have been shown to reduce tissue damage in various disease situations, including ischemia, neuronal apoptosis, and chemotherapy-induced ovary dysfunction [5–8]. In animal studies, ASCs differentiated into osteoblasts *in vitro* and assisted bone formation after being injected *in vivo* under the skin in rats [9]. ASCs injected into damaged periodontal tissue give rise to alveolar bone, cementum, and periodontal ligaments in Wistar rats [10]. In clinical studies, autologous ASC infusion reversed after traumatic calvarial defects in a 9-year-old girl [11] and promoted the healing of fistulae associated with Crohn’s disease [12]. Transplant of ASC-enriched fat grafts, defined as cell-assistant lipotransfer, showed better therapeutic effects compared with fat injection alone in the repair of soft-tissue defects resulting from tumor resection, trauma, and burns [13, 14].

ASCs have been tested in the treatment of diabetes via multiple approaches. First, ASCs have directly differentiated into insulin-producing cells through a multiple-stage differentiation protocol *in vitro* [15–20]. Differentiated cells stained positive for dithizone and expressed pdx-1, c-peptide, insulin, glucagon, and other β-cell markers as well as leptin and adiponectin, which reflected their adipose tissue origins. Insulin production was observed when ASC-derived insulin + cells were transplanted into streptozotocin (STZ)-induced diabetic mice, although the amount of insulin secreted was relatively low compared with insulin secreted by mature pancreatic islets [21].

Second, based on their anigogeneic, anti-apoptotic, and anti-inflammatory properties, ASCs have been co-transplanted with islet grafts to improve graft survival after transplantation. For example, co-transplantation of allogeneic mouse islets with autologous ASCs under the kidney capsule prolonged allogeneic islet survival in mice [22]. Co-encapsulation of pig islets with ASCs improved oxygenation, neoangiogenesis, and the long-term function of a subcutaneous of transplanted islets in a preclinical primate islet transplantation model [23]. Implantation of ASCs and adipose tissue enhanced subcutaneous grafting of islets in diabetic mice by contributing to islet graft survival and revascularization after transplantation in diabetic mice [24]. Islets co-transplanted with ASCs pre-treated with a mixture of hyaluronic, butyric, and retinoic acid manifested enhanced islet revascularization in diabetic rats [25]. In patients with type 1 diabetes, co-transplantation of ASC-derived insulin-secreting islets with hematopoietic stem cells decreased exogenous insulin requirement, increased c-peptide levels, and prevented ketoacidosis [26]. In another clinical trial in the same patient population, transplantation of ASC-derived insulin^+^ cells improved patients’ HB1Ac levels, and decreased serum GAD antibody, without causing adverse effects [27].

Third, intravenous injection of ASCs showed efficacy in reducing hyperglycemia in various diabetic mouse models. For example, in STZ-induced diabetic models, injection of ASCs ameliorated fasting blood glucose and pancreatic islet damage and improved insulin generation in Sprague-Dawley rats [28] and C57BL/6 mice [29]. In the spontaneous non-obese diabetic (NOD) mouse model, a single injection of ASCs reversed hyperglycemia associated with early-onset diabetes in 78 % of NOD mice, by regulation of Th1-biased immune response, expansion of regulatory T cells (Tregs), and reduction of inflammatory cell infiltration in the pancreas [30].

In addition, ASC infusion showed therapeutic effects in the treatment of diabetes-related complications. For example, ASCs from humans or rats ameliorated diabetic retinopathy in diabetic rats [31, 32] and protected podocytes from high-glucose-induced apoptosis [33]. Stage-specific embryonic antigen-3-positive ASCs accelerated wound healing associated with type 1 diabetes [34].

The goal of this study was to determine the therapeutic effects and mechanisms of action of ASCs in restoring glucose homeostasis in DIO mice. In this study, we injected a single dose of ASCs into DIO mice and assessed the impact of this injection on mouse glucose disposal and insulin sensitivity. Our goal was to gain insight into the mechanisms of ASC therapy. We speculate that the tissue-repairing property of ASCs contributed to their therapeutic effects in the DIO mice.

## Methods

### Animals

Male C57BL/6 mice (6 weeks old) and Tg(CAG-EGFP)B5Nagy transgenic mice in which the entire mouse organ system expresses GFP were purchased from Nanjing Biomedical Research Institute of Nanjing University (Nanjing, China) and allowed to adapt to the new environment for 1 week. At 7 weeks of age, mice were fed with either a high-fat diet (60 % of calories from fat) or a standard CHOW-fat diet (10 % of calories from fat) for 20 weeks before treatment was performed as in a previous study [35]. All animal experiments had been approved by the Institutional Animal Care and Use Committee at Qingdao Agricultural University.

### ASC collection and characterization

Epididymal fat was dissected from healthy C57BL/6 mice fed with normal CHOW washed with phosphate-buffered saline (PBS) and cut into small pieces. Tissues were then digested with collagenase type 1 (Sigma-Aldrich, St. Louis, MO, USA) in PBS and incubated in a shaker at 37 °C for 15–30 minutes. At the end of digestion, 10 % fetal bovine serum (FBS) was added into the mixture to neutralize collagenase. The mixture was then centrifuged at 1200 revolutions per minute (rpm) for 5 minutes to remove floating adipocytes and liquids. Pellets were re-suspended with Dulbecco’s modified Eagle’s medium/F12 medium containing 10 % FBS, 1 % penicillin and streptomycin, and cultured in 37 °C at 5 % CO_2_ atmosphere. Cells were split every 7–10 days. Expression of cellular markers (CD105, CD44, CD29, CD14, and CD31) was determined by flow cytometry analysis. ASCs were induced to differentiate into adipocytes, osteocytes, and chondrocytes by using cell differentiation kits from R&D Systems (Minneapolis, MN, USA) in accordance with the recommendation of the manufacturer. Adipogenic differentiation was determined by Oil Red O staining. Osteogenic differentiation was determined by Alizarin Red staining. Chondriagenic differentiation was determined by Toluidine Blue staining as described [36]. GFP^+^ASCs were harvested from Tg(CAG-EGFP)B5Nagy transgenic mice and grown *ex vivo* and used to study migration of injected ASCs. All other reagents were from Life Technologies (Carlsbad, CA, USA) unless otherwise stated.

### ASC infusion

Mice were put into a mouse restrainer. Cells at passage 3–4 were trypsinized, counted, and re-suspended in PBS at 1 × 10^7^ cells/ml, and 0.2 ml of cell suspension was slowly infused into the tail vein of each mouse. Control mice received 0.2 ml of PBS at the same injection speed.

### Monitoring of mouse behavior, body weight, and blood glucose levels

Mouse behaviors, including food intake, drinking, licking, and other activities, were observed daily after treatment. Food intake was measured during a 24-hour period every week. Random blood glucose levels (non-fasting) were measured by using a drop of whole blood from a tail incision by using a Sannuo glucometer (Sannuo, Changsha, China). Body weights were measured after blood glucose measurement at around 9 a.m., two or three times per week.

### Intraperitoneal glucose tolerance and insulin tolerance test

For intraperitoneal glucose tolerance, mice were fasted for 16 hours and injected with 2 g/kg of glucose (intraperitoneal). For insulin tolerance test (ITT), mice were fasted for 5 hours and then injected with one dose of insulin at 0.75 U/kg (intraperitoneal; Eli Lilly and Company, Indianapolis, IN, USA). At 0, 15, 30, 60, 90, and 120 minutes after glucose or insulin injection, a drop of blood was drawn from the tail vein, and blood glucose levels were measured.

### Tissue harvesting, serum preparation, and blood biochemistry

At 6 weeks after cell infusion, whole blood was collected into a heparinized tube. Plasma was separated from whole blood by centrifugation at 3500 rpm for 15 minutes and stored at 80 °C for further analysis. Serum triglyceride (TG), high-density lipoprotein (HDL), and insulin were measured by using specific reagent kits (Sigma-Aldrich). For mouse tissue collection, livers and epididymal fat tissue were dissected and weighed. Half of the tissue was cut into small pieces and snap-frozen in liquid nitrogen for further analysis. The other half was fixed in 4 % formalin for histological analysis.

### Real-time polymerase chain reaction analysis

Total RNA was extracted from liver and pancreas tissues by using RNeasy Kit (Qiagen, Venlo, The Netherlands). RNA was converted into cDNA by reverse transcription. Expressions of InsR, PPARγ, IL-6, F4/80, TNF-α, and NOD2 were quantified by reverse transcription-polymerase chain reaction (RT-PCR) analysis as described previously [37]. Beta-actin expression was quantified in each sample and used as an endogenous control. Real-time RT-PCR primers were purchased from Life Technologies (Invitrogen Trading Co., Ltd., Shanghai, China).

### Hematoxylin-and-eosin staining

Hematoxylin-and-eosin (H&E) staining was performed as described in a previous study [35]. Fixed tissues were embedded in paraffin and sectioned into slices of 5 μM each. Slides were stained in hematoxylin for 6 minutes, rinsed with water, and stained with eosin for another 1–2 minutes. Sections were dehydrated in 50 %, 70 %, 80 %, 95 %, and 100 % alcohol solutions, cleared with xylene, and mounted with a cover slip onto a labeled glass slide. Slides were observed by using an Olympus BX51 microscope (Olympus, Tokyo, Japan), and images were captured by using an Olympus DP72 digital camera. Diameters of individual fat cells (n = 200 in each group) were calculated by using CellSens Standard software (Olympus).

### Measurement of pancreatic β-cell mass

Whole pancreas tissue fixed in paraffin was continuously sectioned at 6 μM of thickness. Tissue sections were collected every 100 μM. About 80–100 sections were collected for each pancreas. Tissue sections were then stained with the anti-insulin antibody by immunohistochemistry. After staining, four sections of the same size were randomly selected in each slide. Insulin + area in each section was calculated, and an average was obtained. For cell-tracking experiments, liver and pancreas tissues from mice that received GFP^+^ ASCs were collected at 7 and 14 days after cell infusion. The presence of GFP^+^ cells was analyzed at mRNA level by RT-PCR analysis or by immunohistochemistry using the anti-GFP antibody.

### Statistical analysis

Unless otherwise stated, data are expressed as mean ± standard deviation. Data were analyzed by using one-way analysis-of-variance and unpaired Student’s *t* tests with Bonferroni correction. *P* values of less than 0.05 were considered statistically significant.

## Results

### Generation of DIO mice

**Fig. 1:**
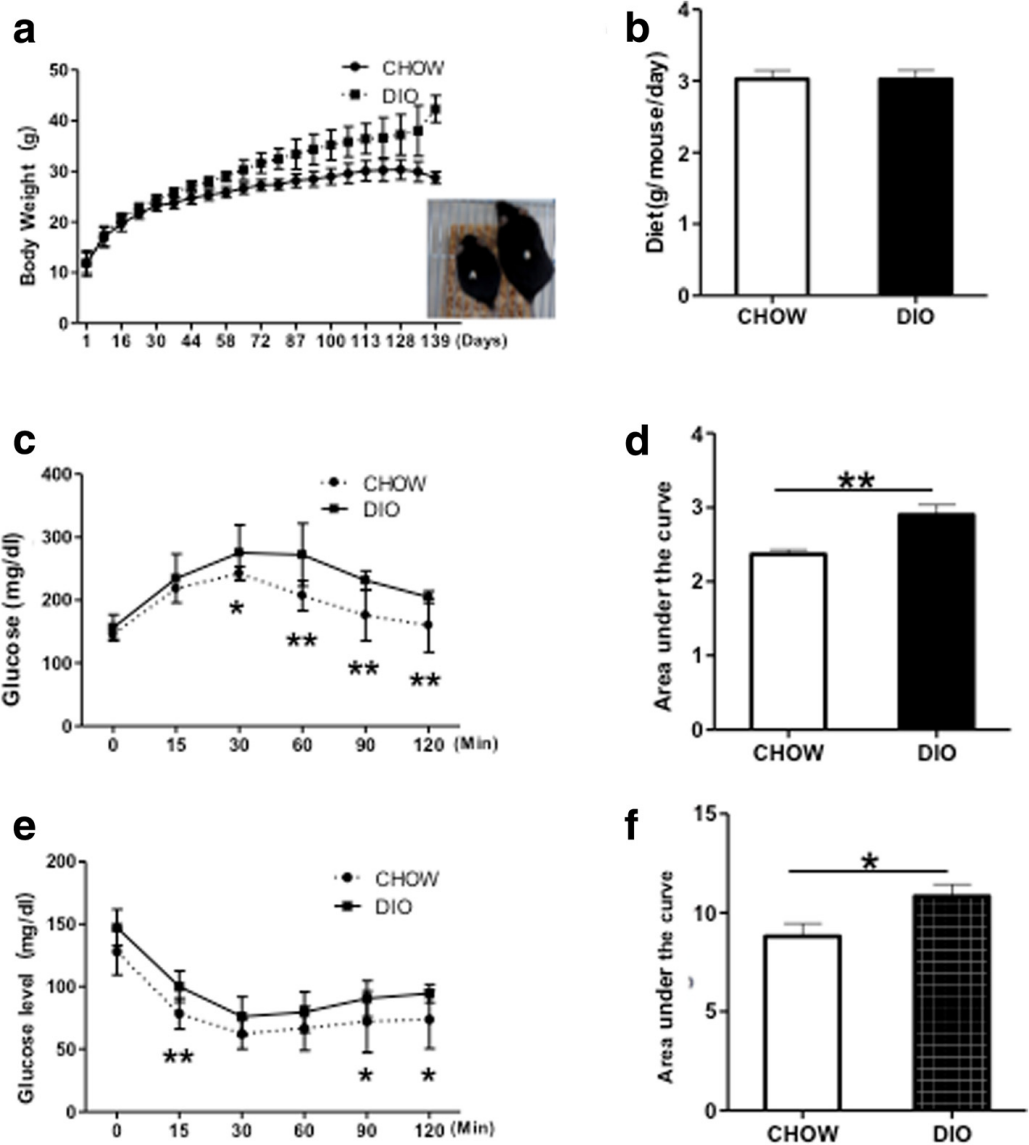
Generation of DIO mice by high-fat diet feeding. **a** Changes in body weights in C57BL/6 mice fed with normal CHOW (CHOW, n = 14) or high fat diet (DIO, n = 37). *Inset*: pictures of CHOW (a) and DIO (b) mice. **b** Average food intake per mouse per 24-hour period (n = 14 in CHOW and n = 22 in DIO). **c** Intraperitoneal glucose tolerance test (GTT) of DIO mice and CHOW controls. **d** Area under the curve of GTT. **e** Insulin tolerance test (ITT) of DIO mice and CHOW mice. **f** Reverse area under the baseline above the curve, n = 5–10 in each group. *P < 0.05; **P < 0.01, analysis-of-variance test. *CHOW* mice fed standard diet, *DIO* diet-induced obese mice treated with vehicle

We generated DIO mice by using a protocol described in a previous study [35]. At 20 weeks after feeding with the high-fat diet, C57BL/6 mice showed significant increase in body weight and reached 42.32 ± 2.63 g, as compared with 28.72 ± 1.2 g in mice fed a standard diet (CHOW, Fig. 1a, Table 1). The average blood glucose level was 170.92 ± 10.31 mg/dl, compared with 126 ± 6.92 mg/dl in CHOW mice (Table 1). No difference in food intake amount was observed between CHOW and DIO mice (Fig. 1b). Furthermore, mice showed impaired glucose disposal after the glucose tolerance test (Fig. 1c and d). DIO mice also had reduced insulin sensitivity after ITT and area under the curve above the basal line (Fig. 1e and f). It seems that we had set up the DIO mouse model with decreased glucose tolerance and insulin sensitivity.

**Table 1:** Body weight and blood glucose of DIO and CHOW mice

Groups	Body weight, g			Blood glucose, mg/dl
Ages	7 weeks	21 weeks	27 weeks	
CHOW	17.11 ± 2.05	27.4 ± 2.97	28.72 ± 1.2	126 ± 6.92
DIO	16.96 ± 1.91	32.48 ± 2.97*	42.32 ± 2.63*	170.92 ± 10.31*

*CHOW* mice fed standard diet, *DIO* diet-induced obese mice treated with vehicle

*P < 0.05 compared with CHOW

### ASC administration reduces body weight and blood glucose levels and ameliorates insulin resistance in DIO mice

**Fig. 2:**
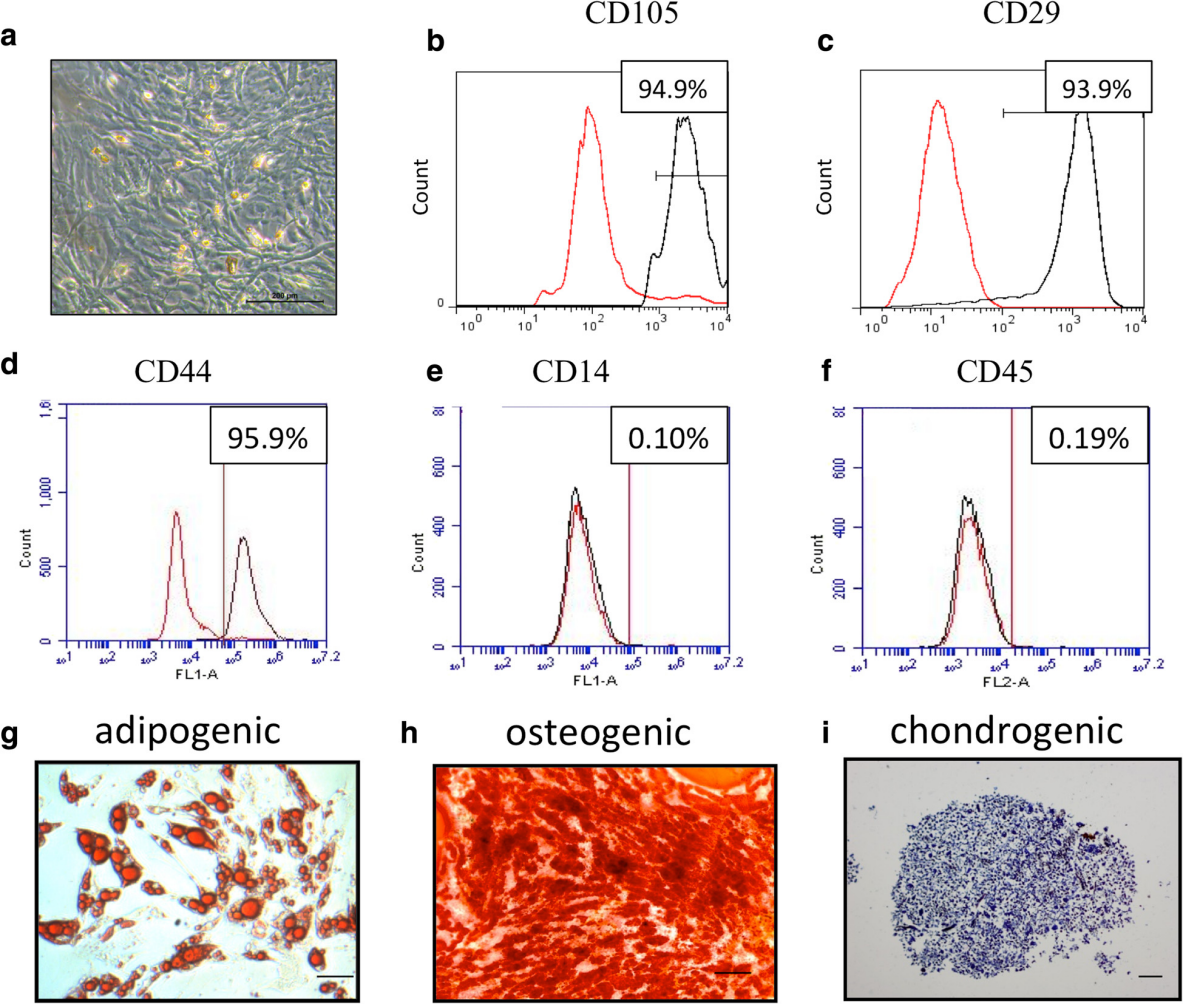
Characterization of ASCs. **a** Micrograph of ASCs at passage 1 under light microscopy. Expression of CD29 (**b**), CD105 (**c**), CD44 (**d**), CD14 (**e**), and CD45 (**f**) in ASCs as measured by flow cytometry analysis. *Red lines* represent cells stained with corresponding isotype control, and *black lines* represent cells stained with individual antibody. Representative micrographs of ASC-derived adipocyte identified by Oil Red staining (**g**), osteocytes by Alizarin Red staining (**h**), and chondrocytes by Toluidine Blue staining (**i**). Bar = 100 μM. *ASC* adipose-derived stem cell

We first characterized ASCs harvested from adipose tissue from C57BL/6 mice fed with normal CHOW. *Ex vivo* expanded ASCs were fibroblast-like and showed normal cell growth as bone marrow-derived MSCs (Fig. 2a). They expressed CD105, CD29, and CD44 and did not express CD14 and CD45 (Fig. 2b–f). They could be differentiated into adipocytes, chondrocytes, and osteocytes as identified by standard differentiation methods (Fig. 2g–i).

**Fig. 3:**
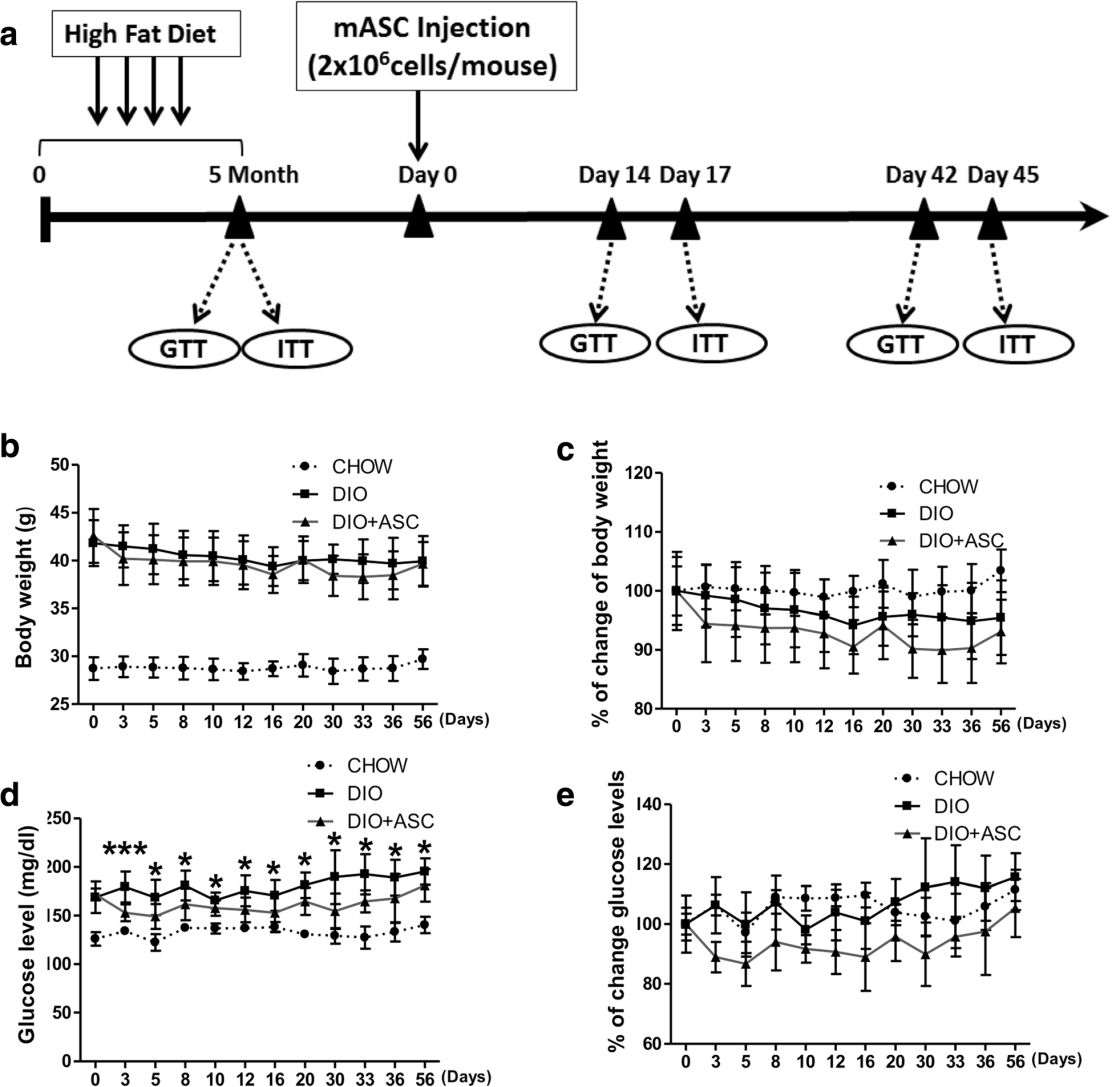
The effects of ASC infusion on blood glucose levels and body weights. **a** Treatment schematics of ASC infusion. **b** Changes in body weights and **c** percentages of body weight changes after ASC injection in CHOW (n = 6), DIO control (n = 6), and DIO + ASC (n = 11) mice. **d** Random (non-fasting) blood glucose levels and **e** percentages in blood glucose change in CHOW, DIO, and DIO + ASC mice. *P < 0.05, **P < 0.01, ***P < 0.01. *ASC* adipose-derived stem cell, *CHOW* mice fed standard diet, *DIO* diet-induced obese mice treated with vehicle

Next, we assessed whether a single intravenous infusion of ASCs could reduce obesity and insulin resistance in DIO mice. As shown in Fig 3a, at 20 weeks after feeding with a high-fat diet, DIO mice received intravenous injections of ASCs at a dose of 2 × 10^6^ cells per mouse. Compared with the body weights of DIO controls, those of DIO mice that had received ASCs were slightly reduced. However, the difference between both groups was not significant (Fig. 3b and c). In contrast, blood glucose levels of ASCs-treated mice were significantly reduced at each time point measured (Fig. 3d and e), indicating an impact of ASC treatment on glucose metabolism in DIO mice.

### ASC injection improves glucose disposal and the effect is persistent

**Fig. 4:**
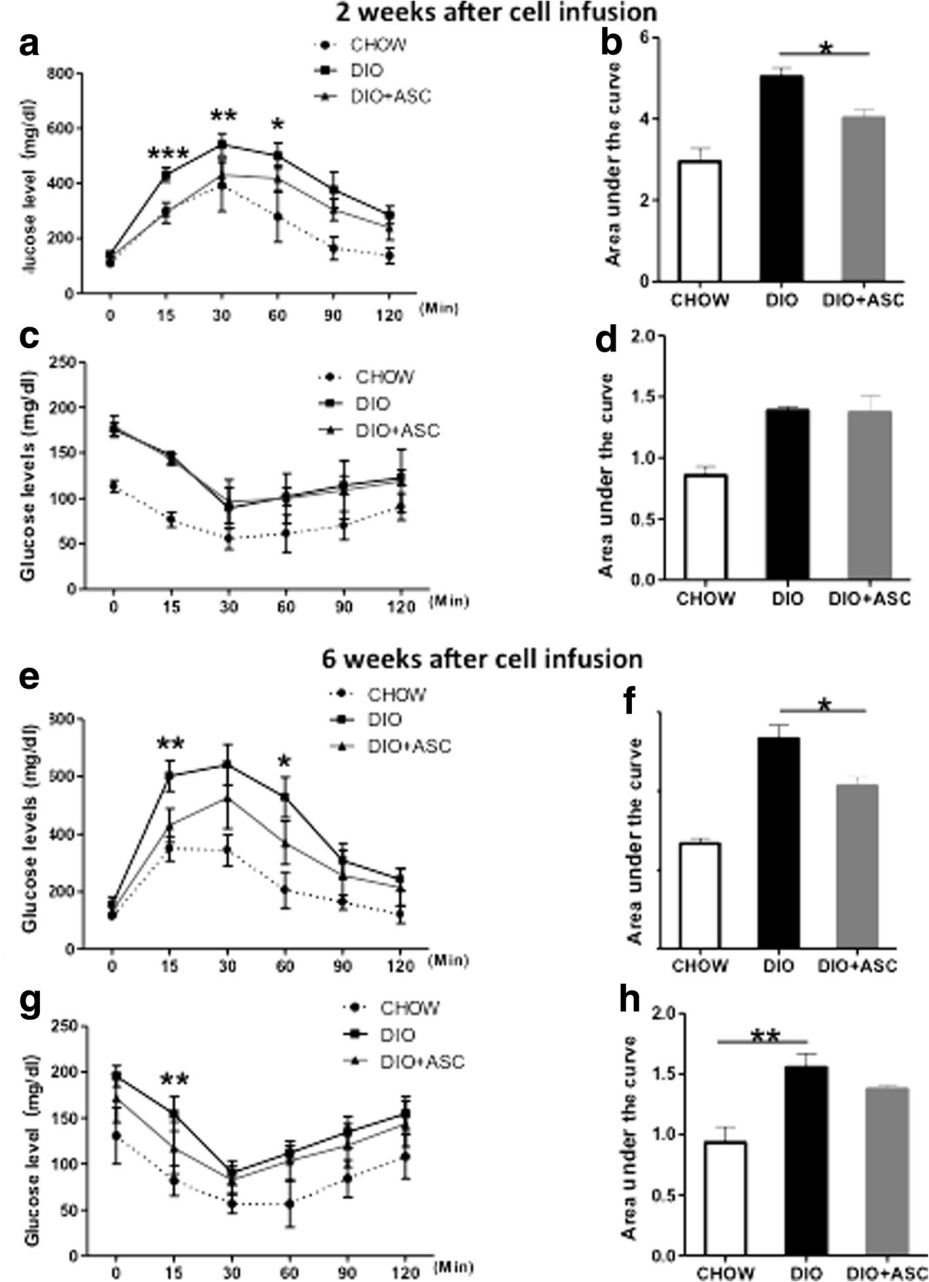
Glucose tolerance and insulin sensitivity in CHOW, DIO, and DIO + ASC mice after treatment. **a** GTT and **b** area under the curve of the GTT test in CHOW, DIO, and DIO + ASC mice 2 weeks after ASC or saline injection. **c** ITT and **d** area under the curve of CHOW, DIO, and DIO + ASC mice at 2 weeks after cell infusion. **e** GTT and **f** area under the curve of GTT test in CHOW, DIO, and DIO + ASC mice 6 weeks after ASC or saline injection. **g** ITT and **h** area under the curve of CHOW, DIO, and DIO + ASC mice at 6 weeks after cell infusion, n = 4–5 in each group. *P < 0.05, **P < 0.01, ***P < 0.01, Student’s *t* test. *ASC* adipose-derived stem cell, *CHOW* mice fed standard diet, *DIO* diet-induced obese mice treated with vehicle, *GTT* glucose tolerance test, *ITT* insulin tolerance test

We next measured the impact of ASC injection on glucose disposal and insulin sensitivity. At 2 weeks after cell infusion, DIO mice showed faster glucose disposal after glucose challenge (2 g/kg, Fig. 4a and b, area under the curve). No difference in insulin sensitivity between the DIO and DIO + ASC groups was observed at this time point (Fig. 4c and d).

To determine whether the effect of ASCs on glucose disposal persists, we performed glucose tolerance test and ITT at 6 weeks after cell infusion. Mice that received ASCs showed faster glucose disposal as demonstrated by significantly lower blood glucoses compared with DIO controls at various times and area under the curve after glucose injection (Fig. 4e and f). In addition, these mice showed slightly better insulin sensitivity, but the difference was insignificant (Fig. 4g and h). These data suggest that a single injection of syngeneic ASCs improved glucose disposal and provided protective effects that persisted long after the treatment had ended. In addition, no significant differences in food intake were observed among CHOW, DIO, and DIO + ASC group throughout the study (data not shown).

### ASC infusion preserved β-cell mass in DIO mice

**Fig. 5:**
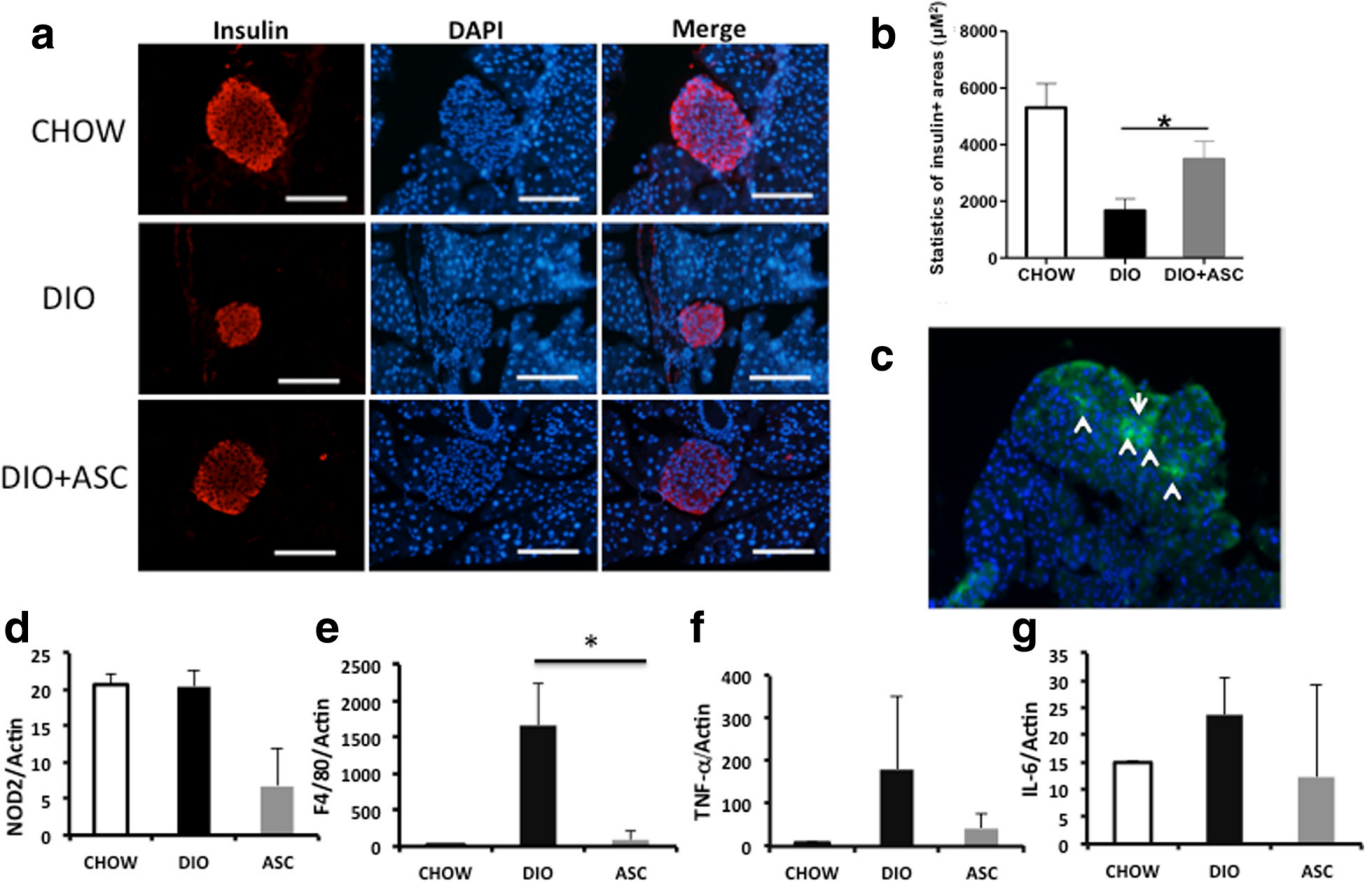
Immunofluoroscent staining of pancreases from CHOW, DIO, and DIO + ASC mice. **a** Representative staining of pancreatic islets from CHOW, DIO, and DIO + ASC mice. Red represents insulin + cells. Blue stains for nuclei. Bar = 100 μm. **b** Statistics of islets/β-cell area in mice from each group. At least three mice were included in each group. **c** Immunofluorescent staining for GFP^+^ cells under fluorescent microscope. Arrows pointed to GFP^+^ cells. Scale bar = 100 μm. At least three mice were analyzed in each group; *P < 0.05, analysis-of-variance test. Relative mRNA expression of *NOD2* (**d**), *F4/80* (**e**), *TNF-α* (**f**), and *IL-6* (**g**) in pancreases of CHOW, DIO, and DIO + ASC (ASC) mice as measured by reverse transcription-polymerase chain reaction analysis. *P < 0.05. *ASC* adipose-derived stem cell, *CHOW* mice fed standard diet, *DIO* diet-induced obese mice treated with vehicle, *NOD2* nucleotide-binding oligomerization domain containing 2, *TNF-α* tumor necrosis factor-alpha

To determine the potential mechanisms of action of ASC injection on glucose disposal, we measured pancreatic β-cell mass in CHOW, DIO, and DIO + ASC mice collected at the end of experiments by staining whole pancreas sections by using the anti-insulin antibody. DIO mice showed significantly reduced pancreatic β-cell area compared with CHOW controls. In contrast, mice that received ASCs showed well-preserved pancreatic β-cell mass comparable to that of CHOW control mice (Fig. 5a and b). These data showed that ASC treatment prevented pancreatic β-cell loss that had been induced by a high-fat diet. MSCs have been shown to migrate to sites of injury, including pancreatic islets after intravenous injection [38, 39]. To determine whether injected ASCs can migrate to the pancreas, we injected GFP^+^ ASCs into mice and measured the presence of GFP^+^ cells after cell infusion. GFP^+^ cells were observed in pancreases at both 7 and 14 days after cell infusion (Fig. 5c). We further measured expression of inflammatory cytokines, including T*NF-α,NOD2*, *F4/80*, and *IL-6*, in pancreases harvested from CHOW, DIO, and DIO + ASC mice. Our data showed elevated mRNA expression of TNF-α, F4/80, and IL-6 in the DIO group compared with mice from CHOW. In contrast, ASC-treated pancreases showed significantly reduced mRNA expression of F4/80 compared with DIO control. Expression of TNF-α was also reduced compared with DIO, but the difference was not significant (Fig. 5d–g). These data suggest that ASC infusion suppressed inflammation in the pancreas, which may have contributed, at least in part, to preserved pancreatic β-cell mass.

### ASC administration reduced adiposity in liver and adipose tissues

**Fig. 6:**
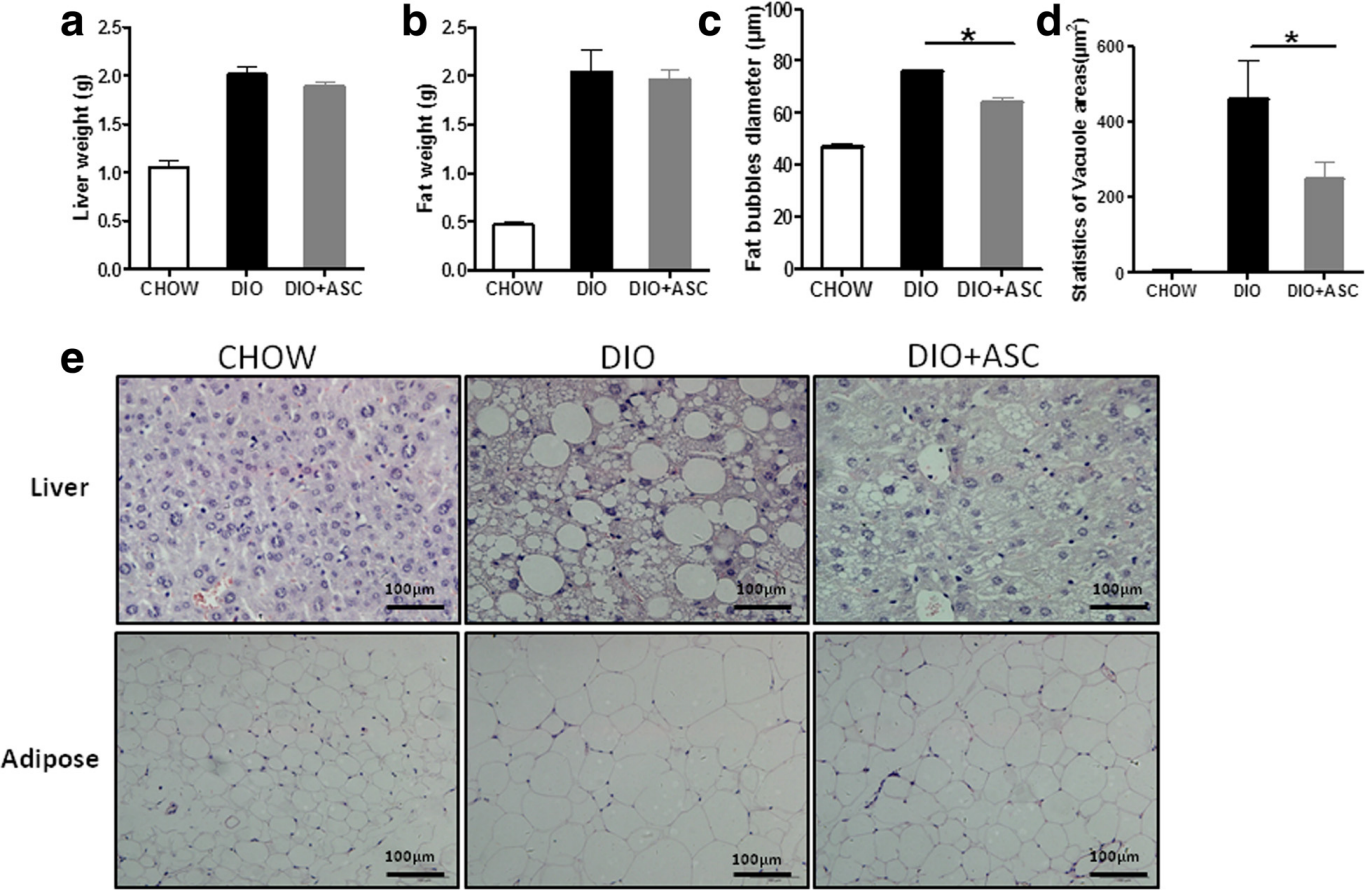
ASC infusion reduces fat liver and adiposity. **a** Liver weights of CHOW, DIO, and DIO + ASC mice. **b** Epididymal fat weights of CHOW, DIO, and DIO + ASC mice. **c** Fat bubble diameters (in micrometers) of epididymal adipocytes of CHOW, DIO, and DIO + ASC mice. **d**. Statistics of vacuole areas in livers of CHOW, DIO and DIO+ASC mice. **e** Representative micrographs of hematoxylin-and-eosin staining of liver and adipose tissue sections from CHOW, DIO, and DIO + ASC mice. *ASC* adipose-derived stem cell, *CHOW* mice fed standard diet, *DIO* diet-induced obese mice treated with vehicle

To determine the effects of ASCs on adipocyte infiltration, liver and epididymal fat tissues were obtained from all mice in each treatment arm at the end of the experiments. No differences in liver and epididymal fat weights were observed between DIO control and DIO + ASC groups (Fig. 6a and b). However, treatment with ASCs improved hepatic steatosis, and fewer fat cells were observed in livers from DIO + ASC mice as demonstrated by vacuole area in liver (Fig. 6d) and by H&E staining (Fig. 6e, upper panel). In adipose tissue, the average diameter of adipocytes in epididymal fat was significantly reduced as well (Fig. 6c and e, lower panel), suggesting that ASC infusion leads to reduced adipocyte size caused by high-fat diet.

### The effect of ASC infusion on TG and HDL expression and liver gene expression

**Fig. 7:**
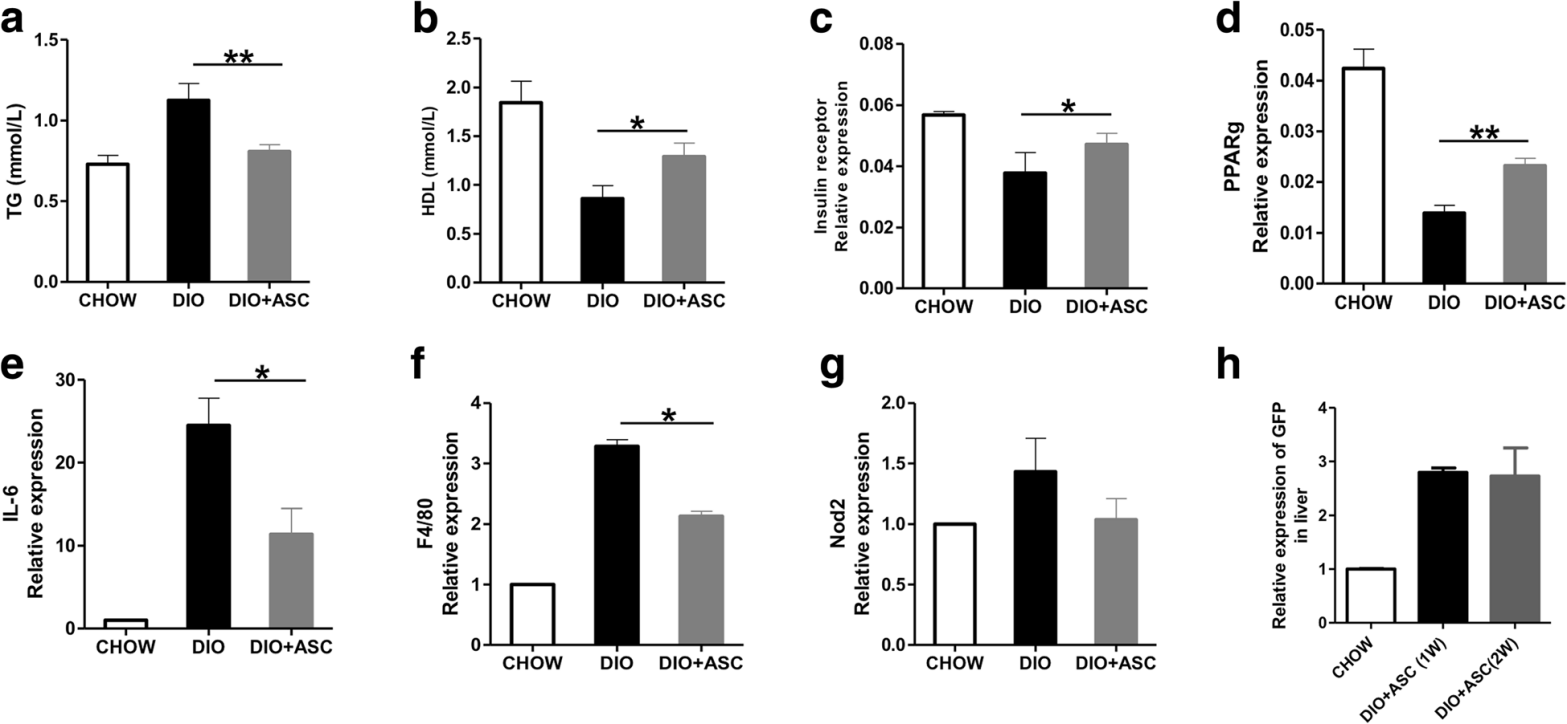
Serum lipid levels and gene expression in livers at 6 weeks after treatment. Serum TG (**a**) and HDL (**b**) levels were measured in CHOW, DIO, or DIO + ASC mice at 6 weeks after ASC infusion. Relative mRNA expression of InsR (**c**), PPARγ (**d**), IL-6 (**e**), F4/80 (**f**), and NOD2 (**g**) in livers of CHOW, DIO, and DIO + ASC mice as measured by reverse transcription-polymerase chain reaction analysis. Values represent relative expression of target gene relative to GAPDH (glyceraldehyde 3-phosphate dehydrogenase) control (National Institutes of Health ImageJ software). Samples from five or six individual mice were analyzed. Data are mean ± standard deviation; *P < 0.05 and **P < 0.01, one-way analysis-of-variance test. (**h**). Relative expression of GFP in livers harvested from CHOW, DIO, and DIO + ASC mice at 7 and 14 days after cell injection. *ASC* adipose-derived stem cell, *CHOW* mice fed standard diet, *DIO* diet-induced obese mice treated with vehicle

We measured serum TG and HDL levels in DIO mice treated with ASCs or vehicle and in CHOW controls. Feeding with high-fat diet in DIO mice showed increased TG level and reduced HDL levels compared with CHOW controls (Fig. 7a and b). ASC treatment significantly reduced TG levels (*P* < 0.01) and increased HDL levels and in DIO mice. High-fat diet causes a reduction in expressions of InsR and PPARγ, an increase in expression of inflammation-related factors (including IL-6), and expression of F4/80 in livers of DIO mice [35]. We measured expression of those genes in CHOW, DIO, and DIO + ASC mice. As is evident in Fig. 7c − g, ASC injection restored expression of InsR and PPARγ and reduced expression of IL-6 and F4/80 expression in livers from DIO mice. There was a reduction of expression of NOD2 in the DIO + ASC group as well. However, the difference was not significant. Furthermore, the presence of ASCs was confirmed in the livers at 7 and 14 days after infusion by measuring mRNA expression of GFP (Fig 7h). These data suggest that one of the potential effects of ASC protection is suppression of inflammation in liver in DIO mice.

## Discussion

The most dramatic finding of this study is that a single infusion of syngeneic ASCs reduced blood glucose levels and improved glucose disposal in DIO mice. The action of ASCs was accompanied by a restored lipid profile (reduced serum TG and increased HDL). In livers, mice in the DIO + ASC group had less fat cell deposition, higher expressions of InsR and PPARγ, and reduced expression of IL-6 and F4/80 compared with DIO control mice. In adipose tissue, DIO + ASC mice had reduced sizes of adipocytes. In pancreases, a well-preserved pancreatic β-cell mass was observed. The protective effects of ASC infusion persisted at least 6 weeks after treatment. One potential explanation for the protective effects of ASCs lies in the suppression of high-fat diet-induced inflammation in insulin-targeting tissues. In obese subjects, macrophages infiltrate into livers and adipose tissue and secrete pro-inflammatory cytokines (including IL-6 and TNF-α) and lead to insulin resistance [40]. We found that fewer fat cell infiltrations in livers in DIO mice received ASCs. At the molecular level, there were reductions in F4/80 and IL-6 and in NOD2 in livers. These data suggest that ASCs improve glucose and insulin sensitivity by suppressing inflammation in insulin-targeting tissue.

Our results provide evidence that one of the potential mechanisms accounting for the protection of ASCs may be their cytoprotective function in pancreatic β cells. Proper function of islets requires sufficient pancreatic β-cell mass and integrity [41]. Beta-cell mass can be reduced by β-cell apoptosis and increased by β-cell replication, neogenesis, or differentiation from stem cells or other cell types. Compared with β-cell mass in healthy individuals, β-cell mass in patients with T2D was decreased to around 60 % with the consequent reduction of glucose-stimulated insulin secretion [42]. Reduced pancreatic β-cell mass in patients with T2D was believed to be due mainly to increased β-cell apoptosis instead of reduction in β-cell replication and neogenesis [43]. We and other investigators [44] have observed increased β-cell mass after bone marrow MSC or ASC injection. However, whether increased β-cell mass was due to reduced β-cell apoptosis or increased differentiation or transdifferentiation from ASCs (or both) is still unclear. Ji et. al showed that both mouse and human insulin-positive cells were found in the pancreases of HFD-fed mice that had received human MSCs, together with detectable human insulin circulating in the blood [44]. Other groups believed that enough pancreatic β cells could not be generated to maintain euglycemia after MSC injection and that cellular differentiation played a minor role in the therapeutic of BM-MSCs [45, 46]. As ASC infusion led to a rapid (3 days after cell infusion) reduction in blood glucose levels in our study, we postulate that one of the major mechanisms of ASC protection observed was due to reduced pancreatic β-cell death. As shown by others [47] and confirmed in our study, ASCs after injection can migrate to an injured pancreas, suppress pro-inflammatory cytokine expression, and contribute to the survival of pancreatic islets. Nevertheless, we do not exclude the possibility that ASCs were indeed differentiated into pancreatic β cells in our study, since we observed a well-preserved pancreatic β-cell mass at 6 weeks after treatment.

In addition, ASC injection promotes expression of molecules related to insulin sensitivity in the liver. As shown in our previous study, expression of InsR and PPARγ was downregulated in livers in DIO mice. We demonstrated in this study that ASC injection restored expression of InsR and PPARγ in livers of treated DIO mouse, indicating that another potential mechanism responsible for the protective effects of ASCs may be exerted via its insulin-sensitizing effects, although we observed only limited improvement in insulin sensitivity in the present study.

Cells infused from an autologous source are considered safer than cells of allogeneic origins [48]. However, it has been shown that diabetes has a negative impact on the therapeutic potential of MSCs. For example, diabetic animals have fewer MSCs, and MSCs’ ability to proliferate and survive in these animals is significantly reduced [49]. MSCs from patients with T2D displayed diminished fibrinolytic activity [50] and defective ischemia recovery [51]. MSCs from T2D mice displayed reduced post-ischemic neovascularization in db/db mice [52]. Considering the above evidence, we used ASCs harvested from healthy C57BL/6 mice in our study. However, it is still worth evaluating whether ASCs from obese or insulin-resistant mice and patients can be used for therapy in clinical application.

## Conclusions

Systemic syngeneic ASC injection reduces blood glucose levels and improves insulin sensitivity in DIO mice. This was associated with suppressed inflammation in the liver and pancreas and well-preserved pancreatic β-cell mass in treated mice. These findings reveal mechanistic insights and provide a new therapeutic strategy for the treatment of obesity, insulin resistance, and T2D.

## Abbreviations

ASC: Adipose-derived mesenchymal stem cell
CHOW: Mice fed with normal chow
DIO: Diet-induced obese mice
FBS: Fetal bovine serum
GFP: Green fluorescent protein
H&E: Hematoxylin and eosin
HDL: High-density lipoprotein
IL-6: Interleukin-6
InsR: Insulin receptor
ITT: Insulin tolerance test
MSC: Mesenchymal stem cell
NOD2: Nucleotide-binding oligomerization domain containing 2
PBS: Phosphate-buffered saline
PPARγ: Peroxisome proliferator-activated receptor γ
rpm: Revolutions per minute
RT-PCR: Reverse transcription-polymerase chain reaction
STZ: Streptozotocin
T2D: Type 2 diabetes
TG: Triglyceride
TNF-α: Tumor necrosis factor-alpha
